# Comparative study of the inhibitory effects of different antibiotic administration routes on bone healing in a rat tibial infection model

**DOI:** 10.3389/fcimb.2025.1529692

**Published:** 2025-02-28

**Authors:** Xiaoyu Han, Wei Wang, Zengli Shen, Lisong Lv, Bingyuan Lin, Haiyong Ren, Yiyang Liu, Qiaofeng Guo, Huang Kai, Xiang Wang

**Affiliations:** Tongde Hospital of Zhejiang Province, Hangzhou, China

**Keywords:** bone infection, antibiotic treatment, rat model, osteomyelitis, bone healing

## Abstract

**Objective:**

This study aimed to evaluate the effectiveness of intravenous versus oral antibiotic treatments in managing bone infections, particularly osteomyelitis, using a rat tibial infection model.

**Methods:**

A tibial bone infection model was established in twelve-week-old Wistar rats via injection of *Staphylococcus aureus* at a cortical defect site. After six weeks, rats were treated with vancomycin (intravenous), cefazolin (intravenous), ciprofloxacin (oral), or ciprofloxacin combined with rifampin (oral). Microbial analysis, blood analysis for pro-inflammatory cytokines, micro-computed tomography (μCT), histological analysis, and osteoclast activity were used to assess the efficacy of each treatment.

**Results:**

Blood analysis showed significant reductions in white blood cell count and pro-inflammatory cytokines in the intravenous treatment groups, especially with vancomycin. μCT imaging revealed better preservation of bone structure in intravenous treatment groups, while oral treatments resulted in more pronounced structural deterioration. Microbial analysis confirmed a lower bacterial load in the intravenous groups, particularly vancomycin, compared to oral treatments. Histological analysis revealed reduced inflammation, lower fibrosis, and minimal bacterial presence in intravenous groups. Osteoclast activity was notably reduced in the vancomycin and cefazolin groups, indicating better control of bone resorption.

**Conclusion:**

Intravenous administration of vancomycin demonstrated superior efficacy in controlling bone infection, reducing inflammation, and preserving bone structure compared to oral treatments. While ciprofloxacin and the ciprofloxacin-rifampin combination showed some efficacy, they were less effective than intravenous vancomycin, likely due to lower bioavailability and insufficient drug penetration in bone tissue.

## Introduction

Bone infections, especially osteomyelitis, are significant complication in orthopedic surgery and pose substantial risks to patient recovery and long-term outcomes (Thabit et al., 2019). Osteomyelitis, characterized by inflammation and destruction of bone tissue, is typically difficult to treat due to the complex anatomy and unique vascular characteristics of bone, which often hinder effective delivery of therapeutic agents to the infection site (Lew and Waldvogel, 2004). Among the various pathogens linked to bone infections, *Staphylococcus aureus* remains the most prevalent and aggressive, capable of forming biofilms on bone surfaces and penetrating deeply into bone tissue (Wen et al., 2020). This biofilm formation contributes to the pathogen’s resilience, making infections more resistant to antibiotic treatment and increasing the likelihood of recurrent infections.

Antibiotic therapy remains the cornerstone of bone infection management, aiming to eradicate the infection while minimizing tissue damage and preserving function (Landersdorfer et al., 2009; Wen et al., 2020). However, effective treatment still faces multiple challenges. Due to the limited blood supply of bones, achieving adequate concentrations of antibiotics at the infection site can be difficult (Li et al., 2019). Additionally, the penetration and retention of antibiotics in bone tissue can vary significantly depending on the type of antibiotic used. For instance, glycopeptides, β-lactams, and fluoroquinolones each have unique distribution properties, half-lives, and efficacy profiles in bone tissue (Masters et al., 2022).

The administration route (e.g., intravenous vs. oral) also greatly impact the drug concentration within the bone. Intravenous administration is often preferred for its rapid and reliable drug delivery (Thabit et al., 2019; Wen et al., 2020), but it requires hospitalization and can be resource-intensive. Conversely, oral antibiotics offer ease of administration and potential for outpatient treatment but may not achieve the same concentration levels in bone tissue as intravenous routes (Sendi et al., 2023). Therefore, identifying the optimal antibiotic and delivery method is critical for improving patient outcomes, reducing treatment duration, and minimizing the risk of drug resistance.

Rat tibial infection models have proven to be valuable tools in evaluating the efficacy of antibiotics in bone infection treatment. In this study, we employed rat tibial bone infection models to investigate the effects of glycopeptides, β-lactams, and fluoroquinolones, administered via intravenous and oral routes, providing data to inform antibiotic selection strategies in bone infection management.

## Materials and methods

### Rat tibial bone infection model construction

Twelve-week-old Wistar rats were used to establish a tibial bone infection model. All procedures were conducted under sterile laminar flow conditions. Anesthesia was administered via intraperitoneal injection of ketamine hydrochloride (80 mg/kg) combined with medetomidine hydrochloride (1 mg/kg). The lateral mid-third of the left hindlimb was shaved, disinfected, and incised to expose the tibial cortex. Using an oscillating saw, a partial cortical defect was created in the tibial midsection. A 20 μL injection of Staphylococcus aureus (strain EDCC 5055, 1×10^3 CFU) was administered at the defect for the model group, while the control group received the same volume of PBS. Bone wax was used to seal the injection site, and the wound was closed in layers.

### Grouping and intervention

Six weeks post-surgery, both the model and control groups underwent a second debridement surgery, involving removal of necrotic tissue, iodine irrigation, and wound closure (Odekerken et al., 2013). Only rats with confirmed bone infection proceeded to intervention groups, divided as follows: Group A (vancomycin, 160 mg/kg, IV every 12 hours), Group B (cefazolin, 160 mg/kg, IV every 12 hours), Group C (ciprofloxacin, 64 mg/kg, oral every 12 hours), Group D (ciprofloxacin + rifampin, 64 mg/kg + 24 mg/kg, oral every 12 hours), Group E (untreated model group), and Group F (normal control group). The sample size of each group was 6 rats. Antibiotic selection and dosing were adapted based on human-rat dose equivalency from clinical guidelines. Antibiotic representatives were selected and used based on a review by Professor Brad, University of California, and the 2015 Infectious Diseases Society of America (IDSA) Clinical Practice Guidelines, with human-rat dose conversion (Spellberg and Lipsky, 2012).

### Sample collection and blood analysis

After the intervention, all rats were euthanized by CO_2_ exposure, and samples were collected under sterile conditions. Blood was immediately drawn from the left ventricle, treated with 0.5 M EDTA, and analyzed using an automated cell counter (Sysmex XT-1800, Dasit, Italy) for white blood cell count, red blood cell count, neutrophil percentage, hemoglobin level, lymphocyte percentage, hematocrit, monocyte percentage, mean corpuscular volume, eosinophil percentage, mean corpuscular hemoglobin content, basophil percentage, absolute neutrophil count, red cell distribution width (coefficient of variation), absolute lymphocyte count, platelet count, absolute monocyte count, mean platelet volume, absolute eosinophil count, absolute basophil count, plateletcrit, platelet distribution width (coefficient of variation), and mean corpuscular hemoglobin concentration. Pro-inflammatory cytokine levels in blood were quantified using ELISA, including macrophage inflammatory protein 2, MIP-2; interleukin-1beta, IL-1β; tumor necrosis factor alpha, TNF-α.

### Micro-computed tomography, μCT

Infected tibias were scanned using μCT, with 3D reconstruction. Bone infection severity graded according to the Odekerken method (Odekerken et al., 2013), which uses a scale from 0 to 4. Grade 0 represents no abnormalities. Grade 1 shows mild periosteal reaction and cortical thickening. Grade 2 includes pronounced periosteal reaction, cortical thickening, and mild osteolysis. Grade 3 is characterized by extensive cortical thickening, focal cortical loss, and marked osteolysis. Grade 4 indicates severe cortical thickening, widespread osteolysis, and complete loss of cortical structure. Bone density (BMD), bone mineral content (BMC), tissue mineral content (TMC), tissue mineral density (TMD), and bone volume fraction (BVF) were quantified with GEHC MicroView 2.0 + ABA software.

### Microbial analysis

For microbial analysis, infected bone tissue was homogenized in PBS containing protease inhibitors cocktail, followed by plating on TSA with 5% sheep blood. Colony-forming units (CFU) of *S. aureus* were quantified and expressed as Log_10_ (CFU/ml).

### Histological analysis

Histological analysis was performed to assess tissue structure and inflammatory response. Decalcified tibial samples were embedded in paraffin, sectioned at 5 μm, and stained with hematoxylin and eosin (HE) to evaluate inflammatory infiltration. Villanueva-Goldner (VG) staining was applied to assess new bone formation. Mineralized nodules were visualized using tartrate-resistant acid phosphatase (TRAP) staining was performed to identify and quantify osteoclasts. Additionally, Gram staining was conducted to detect bacterial presence and distribution within the tissue sections.

### Statistics

Group differences were compared using one-way ANOVA, with data presented as box plots. *Post hoc* comparisons were conducted using Tukey’s HSD test if ANOVA results were significant. Statistical analyses were performed using PASW Statistics 18 (SPSS, USA), with a significance level set at *P* < 0.05.

## Results

### Blood analysis

**Figure 1:**
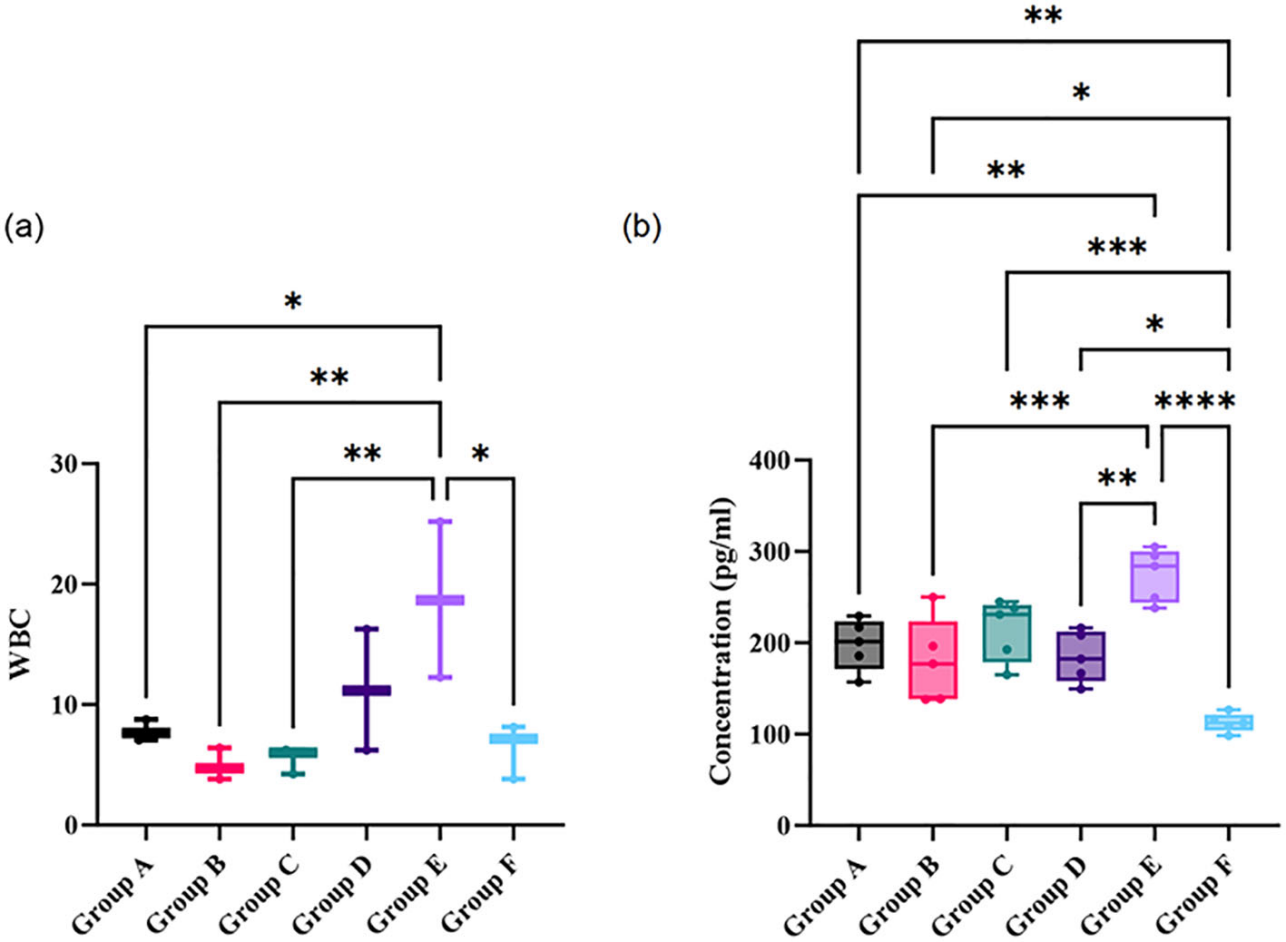
Comparison of white blood cell count **(a)** and concentrations of pro-inflammatory cytokines in peripheral blood **(b)** across different treatment and control groups. Statistical significance between groups is indicated by asterisks: *p < 0.05, **p < 0.01, ***p < 0.001, and ****p < 0.0001. Group A: Intravenous Vancomycin; Group B: Intravenous Cefazolin; Group C: Oral Ciprofloxacin; Group D: Oral Ciprofloxacin + Rifampin; Group E: Untreated Bone Infection Model; Group F: Normal control.

The blood analysis results of each group indicated that only the white blood cell count showed a significant difference among groups (**Figure 1a**; P < 0.05). Specifically, significant differences in white blood cell count were observed between Group A (Intravenous Vancomycin) and Group B (Intravenous Cefazolin), Group A and Group C (Oral Ciprofloxacin), Group D (Oral Ciprofloxacin + Rifampin) and Group B, as well as Group C and Group E (Untreated Bone Infection Model). Notably, the white blood cell count in Group B was significantly higher than in Group A (P < 0.05) and Group D (P < 0.01), while the count in Group E was significantly higher than in Group A (P < 0.01) and Group C (P < 0.05).

Furthermore, the concentration levels of pro-inflammatory cytokines in peripheral blood showed significant differences among the groups (**Figure 1b**; *P* < 0.05). The cytokine concentrations in the bone infection model group were significantly higher than those in the Vancomycin intravenous group, the Cefazolin intravenous group, and the Ciprofloxacin oral group (all *P* < 0.05). Meanwhile, the cytokine levels in the normal control group were significantly lower than those in the other groups (all *P* < 0.05). Notably, there was no significant difference between the Ciprofloxacin oral group and the bone infection model group, suggesting that the anti-infection effect was not evident.

### μCT

**Figure 2:**
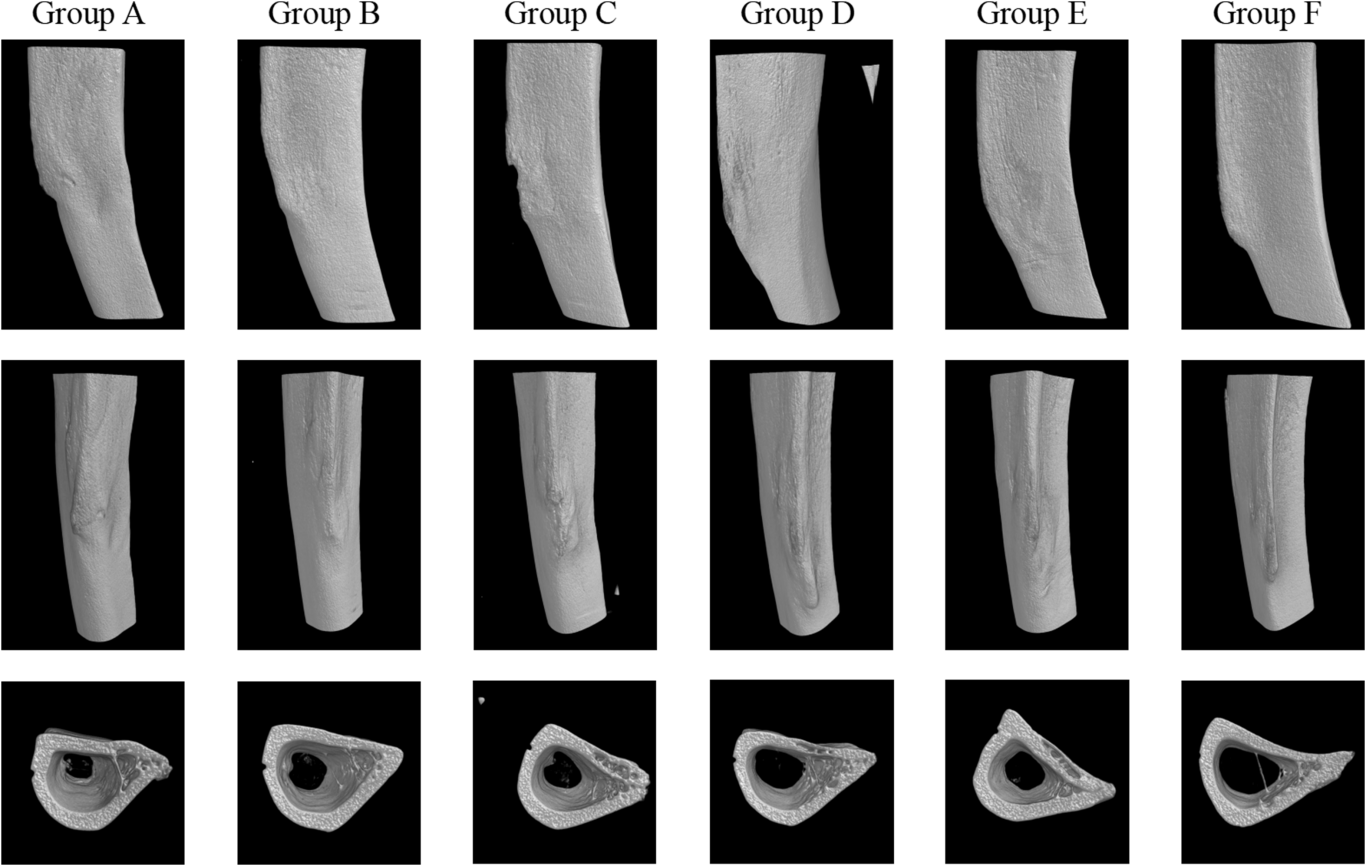
3D reconstructions of groups. Group A: Intravenous Vancomycin; Group B: Intravenous Cefazolin; Group C: Oral Ciprofloxacin; Group D: Oral Ciprofloxacin + Rifampin; Group E: Untreated Bone Infection Model; Group F: Normal control.

The 3D reconstructions showed noticeable structural differences among the groups (**Figure 2**), particularly in the bone infection model group, which shows more pronounced irregularities compared to the other groups, suggesting potential degradation due to infection. While the treatment groups—Vancomycin, Cefazolin, Ciprofloxacin, and Ciprofloxacin + Rifampin—display some preservation of bone structure, none appear to completely restore the morphology to a state comparable to the normal control group. The BMD and TMD analysis shows no significant differences across the groups (both *P* > 0.05).

### Microbial analysis

The data from both the images and the table illustrate clear differences in bacterial load across the various treatment and control conditions. The bone infection model group, which received no treatment, shows the highest bacterial counts, with colony-forming units reaching approximately Log_10_(CFU/ml) values of 6.24, 6.22, and 6.22. In contrast, the normal control group, which was not infected, shows zero bacterial colonies across all samples.

**Figure 3:**
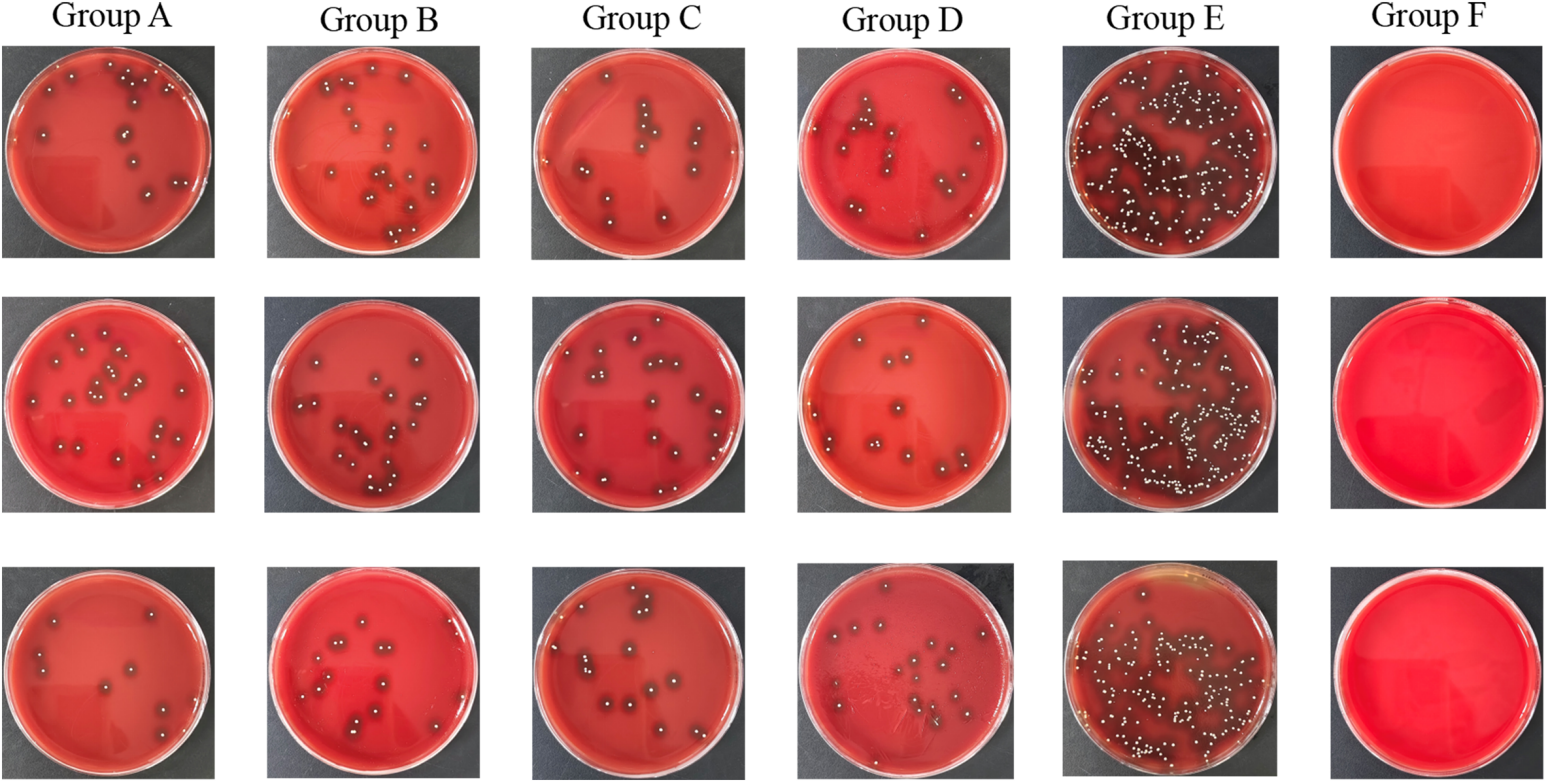
TSA plates of groups. Group A: Intravenous Vancomycin; Group B: Intravenous Cefazolin; Group C: Oral Ciprofloxacin; Group D: Oral Ciprofloxacin + Rifampin; Group E: Untreated Bone Infection Model; Group F: Normal control.

The CFU analysis revealed significant differences between treatment groups (**Figure 3**; *P* < 0.05). The untreated infection model (Group E) exhibited the highest bacterial load (Log~10~ CFU/ml: 6.24 ± 0.12), significantly exceeding all treatment groups (*P* < 0.001 vs. Groups A–D). Intravenous vancomycin (Group A: 5.28 ± 0.24) and cefazolin (Group B: 5.32 ± 0.09) showed comparable reductions (*P* = 0.82 for A vs. B). Oral ciprofloxacin (Group C: 5.31 ± 0.06) and ciprofloxacin+rifampin (Group D: 5.28 ± 0.07) were less effective than intravenous groups (*P* < 0.05 for A vs. C; *P* < 0.01 for A vs. D). No significant difference was observed between oral monotherapy and combination therapy (*P* = 0.75 for C vs. D).

The bacterial colony images on TSA plates further support these findings. The untreated bone infection model displays dense bacterial colonies, reflecting high bacterial titers, while the normal control remains free of colonies. Each of the treatment conditions shows a moderate number of colonies, indicating that while the antibiotics significantly reduce bacterial presence, they do not completely eradicate it.

### Histological analysis

**Figure 4:**
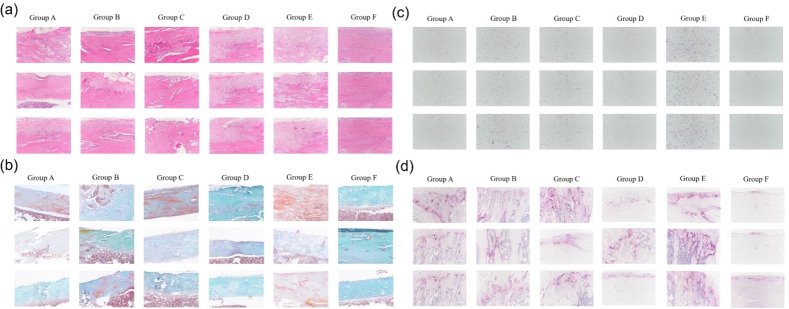
Histological staining of bone tissue sections from each group. **(a)** Hematoxylin and Eosin (HE) staining; **(b)** Villanueva-Goldner’s trichrome (VG) staining; **(c)** Gram staining for bacterial presence; **(d)** Tartrate-resistant acid phosphatase (TRAP) staining to assess osteoclast activity. Group A: Intravenous Vancomycin; Group B: Intravenous Cefazolin; Group C: Oral Ciprofloxacin; Group D: Oral Ciprofloxacin + Rifampin; Group E: Untreated Bone Infection Model; Group F: Normal control.

In the HE staining, nuclei appeared blue, and the cytoplasm displayed a light pink color (**Figure 4a**). The normal control group (Group F) exhibited typical bone structure with no noticeable infiltration of inflammatory cells. In contrast, the bone infection model group (Group E) showed significant tissue destruction and marked infiltration of inflammatory cells, indicating a pronounced infection and inflammatory response. Among the antibiotic treatment groups, inflammation appeared partially reduced, particularly in the intravenous A group (vancomycin) and intravenous B group (cefazolin). In contrast, the oral groups demonstrated a relatively higher degree of inflammatory response, especially in the ciprofloxacin + rifampin group (Group D), implying that oral antibiotic treatment may be less effective.

Villanueva-Goldner’s trichrome (VG) staining revealed the distribution of collagen fibers, which helps assess tissue repair and fibrosis (**Figure 4b**). In the normal control group, collagen fibers were evenly distributed with a clear structure. Conversely, the bone infection model group exhibited significant fibrotic proliferation. The intravenous A group (vancomycin) and intravenous B group (cefazolin) showed lower levels of fibrosis compared to the model group, suggesting a reduction in tissue damage. In contrast, the oral groups, particularly the ciprofloxacin + rifampin group, displayed noticeable fibrotic proliferation.

Gram staining was employed to detect the presence of bacteria (**Figure 4c**). In the normal control group, bacteria were nearly absent, while the bone infection model group showed a substantial presence of bacteria, confirming successful infection induction. The intravenous antibiotic groups (vancomycin and cefazolin) displayed a significant reduction in bacterial count, with the vancomycin group showing almost no detectable bacteria. In the oral groups, some bacteria were still present, especially in the ciprofloxacin + rifampin group.

TRAP staining was used to assess osteoclast activity, reflecting bone resorption levels (**Figure 4d**). The normal control group exhibited low osteoclast activity, consistent with a normal bone metabolic state. In the bone infection model group, osteoclast activity was significantly elevated. Among the antibiotic treatment groups, the intravenous A (vancomycin) and B (cefazolin) groups showed a reduction in osteoclast activity. In contrast, the oral groups, particularly the ciprofloxacin + rifampin group, maintained high osteoclast activity, indicating that the infection was not fully controlled and that bone resorption remained evident.

Quantitative scoring of HE-stained sections demonstrated significantly reduced inflammation in intravenous groups (Group A: 1.8 ± 0.4; Group B: 2.1 ± 0.3) compared to oral groups (Group C: 3.2 ± 0.5, P < 0.01 vs. A; Group D: 3.5 ± 0.6, P < 0.001 vs. A) and the untreated model (Group E: 4.7 ± 0.3, P < 0.001). VG-stained fibrosis scores mirrored these trends: Group A (1.5 ± 0.2), Group B (1.7 ± 0.3), Group C (3.0 ± 0.4, P < 0.01 vs. A), Group D (3.3 ± 0.5, P < 0.001 vs. A), and Group E (4.5 ± 0.2, P < 0.001).

Gram staining confirmed lower bacterial presence in intravenous groups (Group A: 0.3 ± 0.1 bacteria/field; Group B: 0.5 ± 0.2) versus oral groups (Group C: 1.8 ± 0.3, P < 0.001; Group D: 1.5 ± 0.4, P < 0.01) and Group E (3.9 ± 0.5, P < 0.001). TRAP-stained osteoclast counts were reduced in Group A (12 ± 3 cells/mm²) and Group B (15 ± 4) compared to Group C (28 ± 5, P < 0.01), Group D (25 ± 4, P < 0.05), and Group E (42 ± 6, P < 0.001).

## Discussion

The present study highlights clear differences in the effectiveness of intravenous and oral antibiotic treatments for managing bone infection. Importantly, within the intravenous administration groups, no significant difference was observed between the therapeutic effects of vancomycin and cefazolin. Overall, intravenous antibiotics, particularly vancomycin, demonstrated superior efficacy compared to oral treatments, as evidenced across blood analysis, μCT imaging, microbial culture, and histological staining. Blood analysis revealed that both white blood cell counts and pro-inflammatory cytokine levels were significantly lower in the intravenous groups compared to the bone infection model and oral groups, indicating a more effective reduction of systemic inflammation. μCT imaging showed better preservation of bone structural integrity in the intravenous groups, although full restoration was not achieved; meanwhile, the oral groups displayed more pronounced structural deterioration, likely due to insufficient infection control. Microbial culture further supported these findings, with significantly reduced bacterial loads in the intravenous groups, particularly vancomycin, compared to the oral groups, which retained moderate bacterial presence. Histological analyses using HE, VG, Gram, and TRAP staining confirmed these trends: the intravenous groups showed less tissue destruction, reduced fibrosis, minimal bacterial presence, and lower osteoclast activity, indicating controlled inflammation and infection. Together, these comprehensive findings suggest that intravenous antibiotic treatment, particularly with vancomycin, is more effective in controlling bone infection and minimizing tissue damage than oral administration.

Several factors likely contribute to these findings, consistent with previous research in the field. The pharmacokinetic properties of intravenous antibiotics allow for higher and more consistent drug concentrations at the site of infection (Paladino et al., 1991; Stengel et al., 2001). Bone tissue, with its limited blood supply and complex architecture, presents significant challenges for effective drug delivery. Intravenous administration bypasses gastrointestinal absorption and first-pass metabolism, resulting in greater bioavailability and deeper tissue penetration. Vancomycin, a glycopeptide antibiotic, is particularly effective against *Staphylococcus aureus*, the predominant pathogen in osteomyelitis, and can achieve therapeutic concentrations within bone tissue when administered intravenously (Fassbender et al., 2013; Muthukrishnan et al., 2019). Its efficacy is well-documented in previous studies, which highlight its ability to disrupt cell wall synthesis and resist biofilm formation, both critical for eradicating bacteria embedded in bone tissue. The lower bacterial load and reduced inflammation observed in the vancomycin group in this study likely reflect these properties, including its strong tissue penetration and targeted action against biofilm-associated infections (Vergidis et al., 2015).

The microbial analysis demonstrated that intravenous vancomycin and cefazolin achieved significantly lower bacterial loads compared to oral regimens (P < 0.05), aligning with their superior pharmacokinetic profiles. Notably, the lack of difference between vancomycin and cefazolin (P = 0.82) suggests comparable efficacy against S. aureus in this model. Oral ciprofloxacin, even when combined with rifampin, failed to match intravenous efficacy (P < 0.01), likely due to suboptimal bone penetration.

Histologically, intravenous antibiotics significantly reduced inflammation (P < 0.01), fibrosis (P < 0.01), and osteoclast activity (P < 0.05), corroborating their systemic anti-inflammatory and localized antimicrobial effects. The persistence of bacteria in oral groups (P < 0.01 vs. intravenous) underscores the challenge of achieving therapeutic bone concentrations via oral administration.

In contrast, Intravenous vancomycin exhibited superior efficacy in reducing bacterial load (Log~10~ CFU/ml: 5.28 vs. 6.24 in untreated, P < 0.001), inflammation (HE score: 1.8 vs. 4.7, P < 0.001), and osteoclast activity (12 vs. 42 cells/mm², P < 0.001) compared to oral therapies. These statistically robust findings (P < 0.05 for all key comparisons) validate intravenous administration as the preferred route for severe osteomyelitis.

Oral antibiotics, particularly ciprofloxacin, have demonstrated limited efficacy in previous studies of bone infections due to relatively lower bioavailability in bone tissue (Stengel et al., 2001). Although fluoroquinolones like ciprofloxacin possess broad-spectrum activity, their absorption and distribution when administered orally may be insufficient to eliminate infections effectively, especially those involving biofilm-producing bacteria. What’s more, Clinical experience and results from a randomized controlled trial support the activity of rifampicin-quinolone combination therapy for orthopedic implant-associated staphylococcal infections (Zimmerli et al., 1998). In our study, the combination of ciprofloxacin with rifampin, which has shown synergistic effects against *S. aureus (* Yang et al., 2018), did show some improvement over ciprofloxacin alone but still fell short of the efficacy seen with intravenous vancomycin. Rifampin’s ability to penetrate biofilms is advantageous; however, when given orally, its effectiveness may still be constrained by lower overall drug concentrations within bone tissue (Shiels et al., 2018; Albac et al., 2023).

Our findings on inflammatory markers align with previous research linking effective infection control to reduced systemic inflammation in osteomyelitis models. Elevated white blood cell counts and pro-inflammatory cytokine levels in the bone infection model and oral treatment groups suggest a sustained immune response to persistent infection. Studies indicate that, when bacterial load is not effectively managed, the immune system remains activated, leading to ongoing production of inflammatory mediators and osteoclast activation (Doi et al., 2022). This prolonged inflammatory state accelerates bone resorption, contributing to further bone degradation and fibrosis (Zhao, 2020).

## Data Availability

The original contributions presented in the study are included in the article/supplementary material. Further inquiries can be directed to the corresponding authors.
